# Supplementary material for: Comparative multiomics analysis of cell physiological state after culture in a basket bioreactor
Source: Sci Rep. 2022 Nov 23;12:20161. doi: 10.1038/s41598-022-24687-4 (PMC9686226; doi:10.1038/s41598-022-24687-4)

27815206:+@NW_023666077.1:27818840:27818941:+@NW_023666077.1:27820201:27820462:+@NW_02366607

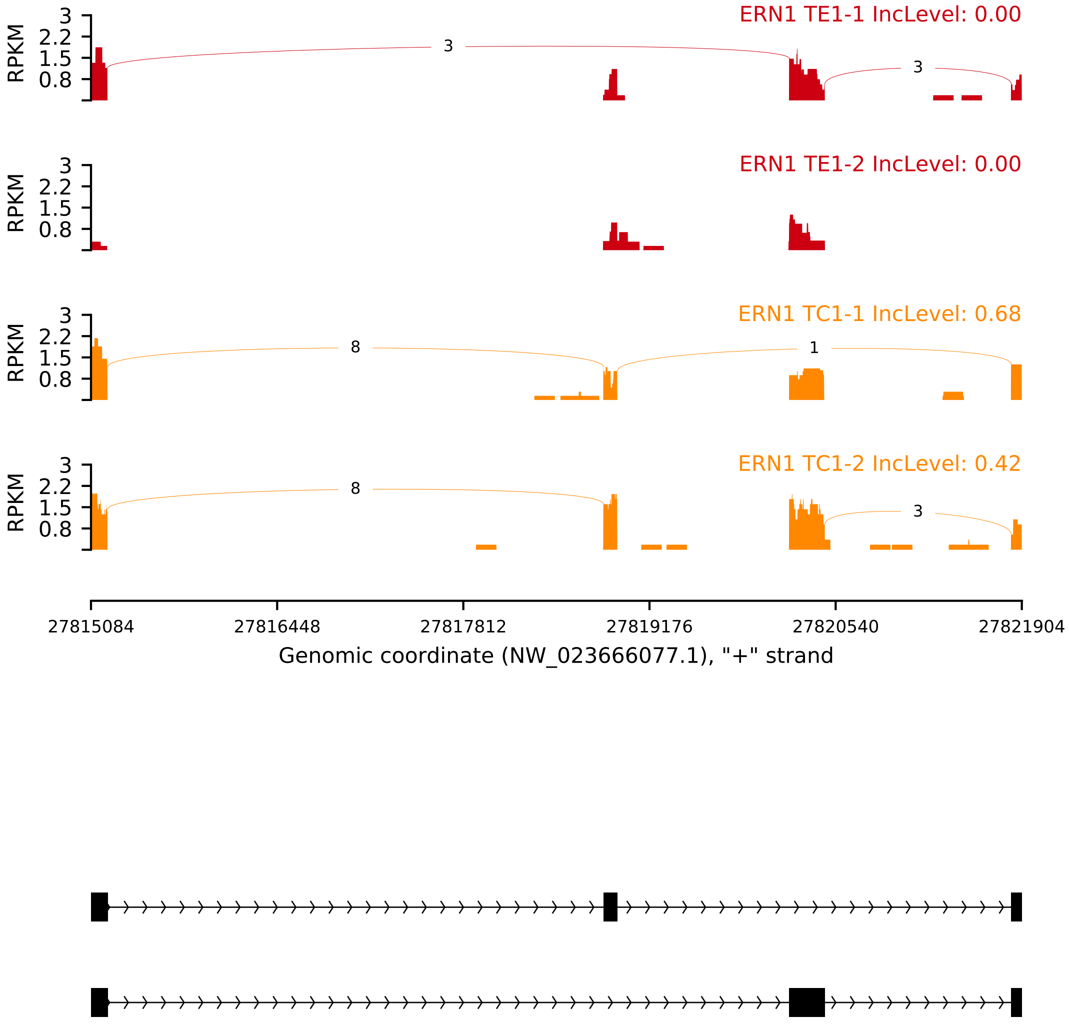

Supplement: Supplementary file 1 — Supplementary Information 1. [file 41598_2022_24687_MOESM1_ESM.zip › raw data/Transcriptomic raw data/7.AS/2.dasplot/TE1vsTC1/MXE/3_ERN1_NW_023666077.1_27815084_27815206_+@NW_023666077.1_27818840_27818941_+@NW_023666077.1_27820201_27820462_+@NW_023666077.1_27821827_27821905_+.pdf]
